# The Power of Technology Addressing Anxiety for a Better Mental Health

**DOI:** 10.1192/j.eurpsy.2022.107

**Published:** 2022-09-01

**Authors:** J.-C. Minier

**Affiliations:** Group GAC, Group Gac, St Brieuc, France

**Keywords:** Mental health technological innovations, blended therapies within the mental health sector

## Abstract

As the manager of a business incubator, I’m involved in the European project IT4Anxiety. It focuses on the creation and implementation of innovative solutions designed to reduce the anxiety of patients experiencing neurological disorders or PTSD. The IT4Anxiety project is a collaboration between mental health professionals, users and start-ups, but also universities, research centres and higher education establishments. There is growing evidence that technological solutions can expand and complement conventional medical protocols. Such “blended therapies” are central to the approach being developed through several work packages. In the framework of the IT4Anxiety project, I carry out actions to develop interactions between startups and mental health professionals.In this context, my speech will focus on 3 specific points: - Feedback on the process of testing and validation of innovative tools in hospitals, care homes… We developped a framework for testing and validating tools with patients and start-ups in order to guarantee the effective implementation of these innovative solutions, especially from an ethical point of view. Going before the ethics committee is often a new experience for start-up managers who have to answer complex questions - Feedback on co-creation processes, in particular through the organisation of hackathons bringing together start-ups, users and health professionals.The objective of the hackathons is to bring together stakeholders in the co-creation of innovative solutions and their integration thereafter into the mental health care sector - Examples of innovative tools in mental health developed by startups which have joined the IT4Anxiety project. These tools complement conventional medical protocols

**Disclosure:**

No significant relationships.

